# Modern drug discovery applications for the identification of novel candidates for COVID-19 infections

**DOI:** 10.1016/j.amsu.2022.104125

**Published:** 2022-07-12

**Authors:** Isha Rani, Avjit Kalsi, Gagandeep Kaur, Pankaj Sharma, Sumeet Gupta, Rupesh K. Gautam, Hitesh Chopra, Shabana Bibi, Syed Umair Ahmad, Inderbir Singh, Manish Dhawan, Talha Bin Emran

**Affiliations:** a MM College of Pharmacy, Maharishi Markandeshwar (Deemed to be University), Mullana, Haryana, India; b MM School of Pharmacy, MM University, Sadopur, Ambala, Haryana, India; c Chitkara School of Pharmacy, Chitkara University-Baddi, Himachal Pradesh, India; d Apotex Research Pvt. Ltd, Bangalore, Karnataka, India; e Department of Pharmaceutics, Chitkara College of Pharmacy, Chitkara University, Punjab, India; f Yunnan Herbal Laboratory, College of Ecology and Environmental Sciences, Yunnan University, Kunming, 650091, Yunnan, China; g The International Joint Research Center for Sustainable Utilization of Cordyceps Bioresources in China and Southeast Asia, Yunnan University, Kunming, 650091, Yunnan, China; h Department of Bioinformatics, Hazara University, Mansehra, Pakistan; i Department of Microbiology, Punjab Agricultural University, Ludhiana, 141004, Punjab, India; j Trafford College, Altrincham, Manchester, WA14 5PQ, UK; k Department of Pharmacy, BGC Trust University Bangladesh, Chittagong, 4381, Bangladesh; l Department of Pharmacy, Faculty of Allied Health Sciences, Daffodil International University, Dhaka, 1207, Bangladesh

**Keywords:** COVID-19, *In silico*, Glycosides, Alkaloids, Computational, Terpenoids

## Abstract

In early December 2019, a large pneumonia epidemic occurred in Wuhan, China. The World Health Organization is concerned about the outbreak of another coronavirus with the powerful, rapid, and contagious transmission. Anyone with minor symptoms like fever and cough or travel history to contaminated places might be suspected of having COVID-19. COVID-19 therapy focuses on treating the disease's symptoms. So far, no such therapeutic molecule has been shown effective in treating this condition. So the treatment is mostly supportive and plasma. Globally, numerous studies and researchers have recently started fighting this virus. Vaccines and chemical compounds are also being investigated against infection. COVID-19 was successfully diagnosed using RNA detection and very sensitive RT-PCR (reverse transcription-polymerase chain reaction). The evolution of particular vaccinations is required to reduce illness severity and spread. Numerous computational analyses and molecular docking have predicted various target compounds that might stop this condition. This paper examines the main characteristics of coronavirus and the computational analyses necessary to avoid infection.

## Highlights

• Importance of advanced genome sequencing in analyzing the sequential change in DNA and RNA due to SARS-CoV-2 genome.
• Lead Identification studies with computer drug-aided design.
• Prevalence of some flavonoid glycosides in medicinal plants which have potential protective effects against COVID-19 infections.
• Docking studies with alkaloids, vanillin derivatives and their actions suggest possible SARS- CoV-2 inhibition.
• Modeling studies outcomes about ligand-based and structure based activity.

## Teaser

Key Highlights:1. Structure and mechanism of action of COVID-19 Virion.2. Advantage of bioinformatics in COVID-19 research.3. CADD as a novel method for discovering phytomedicines for COVID-19.4. Discussion of computational chemistry of various alkaloids, glycosides and terpenoids for COVID-19 treatment.

## 1 Introduction

Coronaviruses are a cluster of viruses that affect animals and humans as effective pathogens. One such novel virus was reported in 2019 as a cause of many pneumonia cases in the province of Hubei, China [1]. Initially, a significant outbreak hit Wuhan City. The arrival of this outbreak brought about tension, attention, and concern from the people across the globe and even the World Health Organization [2]. Since its inception and widespread across different countries, it has evolved and grown exponentially [[3], [4], [5]]. The WHO announced it as a public health emergency primarily of global concern [6]. Coronavirus disease 2019, i.e., now commonly named COVID-19, is caused by a virus known as acute respiratory syndrome coronavirus 2 (SARS-CoV-2) that leads to severe respiratory problems like pneumonia and lung failure [7]. It is believed that it may have evolved from the zoonotic coronaviruses, like SARS-CoV, in 2002. This serious illness started in China and for quite a few times has hit the enormous human casualties leading to a vast global threat and disease of pandemic level [8]. The virus has an incubation period of about 1–14 days. The disease generally initiates with fever, cough, difficulty breathing, and chest stiffness that lasts about 2–3 days [9]. Some people might develop fatal complications like a failure of organs like kidney, liver, septic shocks, disease like pneumonia, pulmonary edema, and sometimes acute respiratory distress syndrome (ARDS) [10]. It is confirmed that the virus originated in bats, but the mediator animal that may have spread it is uncertain.

**Fig. 1:**
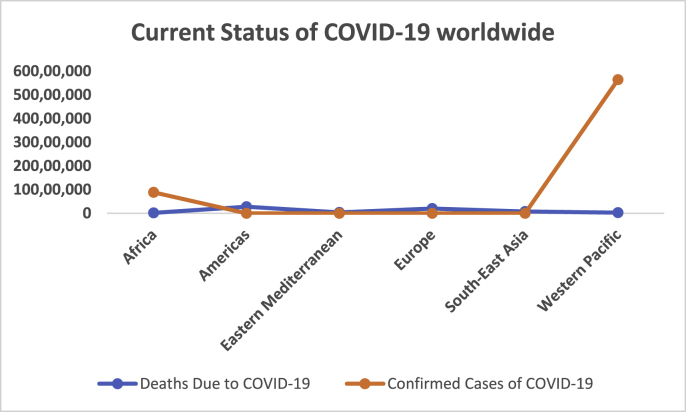
Current status of COVID-19 around the world until May 12, 2022.

The virus's spread needs to be halted, and various techniques of quarantining and treatment should definitely evolve. Also, different strategies are urgently required to cope up with such a problem. Presently, various diagnostic kits are available to test for the virus, alcohol-based sanitizers and filter masks are also being used to contain the spread of this virus. Several medicated therapeutics have been successfully tested clinically effective against the virus. Also, the use of antivirals and symptomatic medications to treat this virus is evolving. Thus, the outbreak of covid 19 has urged various organizations and companies to develop vaccines and different treatment strategies to prevent the worldwide expansion of this virus [11,12]. The pathogen of this disease is a single-stranded RNA virus with a diameter of about 80–120 nm with a spike-like guess on its face [13]. These projections are considered the reason for the virus giving a crown-like appearance under the observation of the electron microscope. Due to such an appearance, it is known and aptly named as the coronavirus. The coronavirus has a circular or elliptic shape [14]. The novel coronavirus class consists of four types of strains such as α-CoV, β-CoV, δ-CoV, and γ-CoV and four strains of this virus, namely HKU1, NL63, 229 E, and OC43, are in circulation in humans, and they are considered to affect the respiratory tract and show mild symptoms [15,16]. There have been two past events of the circulation of such strains that have been due to the crossover of beta coronavirus found in animals to humans resulting in a mild effect towards the respiratory tract. One such instance occurred in 2002–2003, during which a new virus of β genera coronavirus had circulated to humans through the intermediate host being palm civet cats in the Guangdong province of China. This virus was quite severe and provocative, affected around 8422 people in China, and caused about 916 mortalities before it was contained and halted [17]. Almost after a decade, in 2012, the strain of the Middle East Respiratory Syndrome Coronavirus (MERS-CoV) originated from bats got spread in Saudi Arabia and was circulated to humans through the dromedary camels as the intermediate host [18,19]. The well-defined structure of the novel coronavirus causing COVID-19 can be correctly evaluated under the electron microscope. Bats are considered to be the natural host of this virus. A recent study estimated 96.3% homology similar to bat-SARS like coronavirus and Bat CoV RaTG13, so bats are considered to be the most potent COVID-19 in humans [20]. Fig. 1 presented the current status of COVID-19 around the world until May 12, 2022, total number of 516,922,683 cases have been reported, and 6,259,945 COVID-19 patients have died around the world (https://covid19.who.int/).

## 2 Mechanism of action of COVID-19

**Fig. 2:**
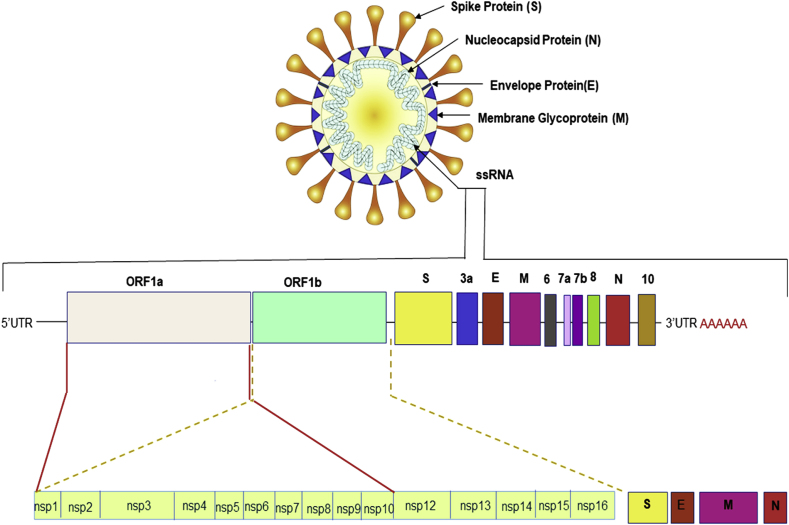
SARS-2/COVID-19 genome structure.

The COVID-19 is very similar to the SARS-CoVs, and the “International Committee on Taxonomy of Viruses” has called it “SARS-CoV-2” (also known as “Corona Virus Study Group 2”). Initially, the coronavirus or SARS-CoV-2 will first attach to an enzyme found in the human body. ACE2 (an enzyme that the virus latches onto with its spike-like projections to infect and enter the other cells in the host's body) is the enzyme the virus uses to gain entry into other cells in the body of the host. The spike-like protein needs to be coagulated or primed by another enzyme called protease (TMPRSS2) to produce additional pathogens after the first step of interacting with the enzyme [21]. After the spike protein has latched on, it is a cellular ligand and is activated by the protease enzyme, and then it enters the host's cell, where it becomes an uncoded protein that uncoats itself. RNA genome transcription occurs in the cytoplasmic membranes, and RNA synthesis occurs in the membrane-bound ribosomes. To make a continuous/non-continuous/complex of proteins-embedded gene-mediated product, the fusion process uses both continuous and non-continuous chemicals and a complex of proteins embedded in a 20-kb replicase gene (As shown in Fig. 2). It is a complex of biological proteins and approximately 16 viral components known as the replicase gene protein complex [22]. This virus includes enzymes such as endoribonuclease, endoribonuclease, 3′-to-5′ exoribonuclease, 2′-O-ribose methyltransferase, ADP ribose 1′-phosphatase, and two groups of coronaviruses: one with cyclic phosphodiesterase activity and another with corona synthesis. Since it was expected that the coronavirus would utilize enzymes to digest different RNAs, RNAs not seen in other RNA viruses were anticipated to be present. Metabolic breakdown causes the formation of mature particles of genomic RNA from broken down proteins that collect in the cell membrane [23,24].

The shape of the virus is spherical. There is a specific lipid coat with a spike glycoprotein on it. SARS-CoV-2 has a normal Betacoronavirus genomic structure. A replicase complex named “ORF1a and ORF1b” is present in the 5′UTR in addition to about 29,903 nucleotides. In the case of ORF1a, nsp1–nsp16 are encoded. Various genes like spike, envelope, membrane, nucleocapsid gene and a polynucleotide tail are attached at 3′UTR. The auxiliary genes flank the structural genes. Reproduced with permission from Ref. [25].

## 3 Modern techniques for COVID-19 drug research

**Fig. 3:**
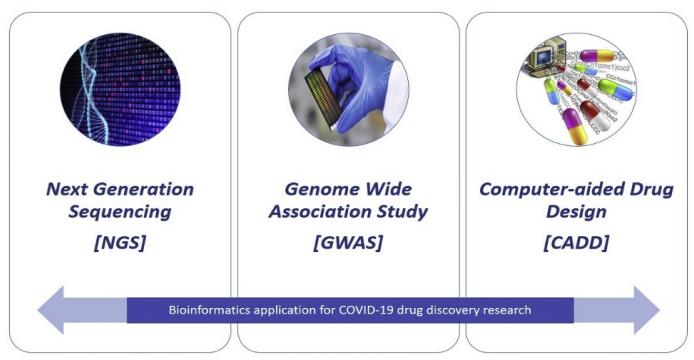
Interconnected approaches of bioinformatics helpful to find novel treatment against COVID-19 infection.

As the scientific community aware of currently most emerged virus SAR-CoV-2, which has several mutated variants inducted in the society now, and becomes the global panademic crisis from last more than two years. It is highly required the novel and unique approaches for the genetic understanding of SARS-CoV-2 and its variants, that has influenced the field of medicinal research, due to which the several novel drugs has been reproposed and also identified from natural sources and potential vaccine candidates have also been discovered to safegaurd the scientific community [26]. Hence, the advantage of bioinformatics in COVID-19 research had created a breakthrough since last few decades [27]. The use of bioinformatics approaches and softwares has successfully understood the SARS-CoV-2 genomic structural design. In silico research involved the major three techniques such as next-generation sequencing, genome-wide association studies, and comuter-aided drug design (CADD) [28] (Fig. 3).

### 3.1 Methods of computer-aided drug design (CADD) for COVID-19

**Fig. 4:**
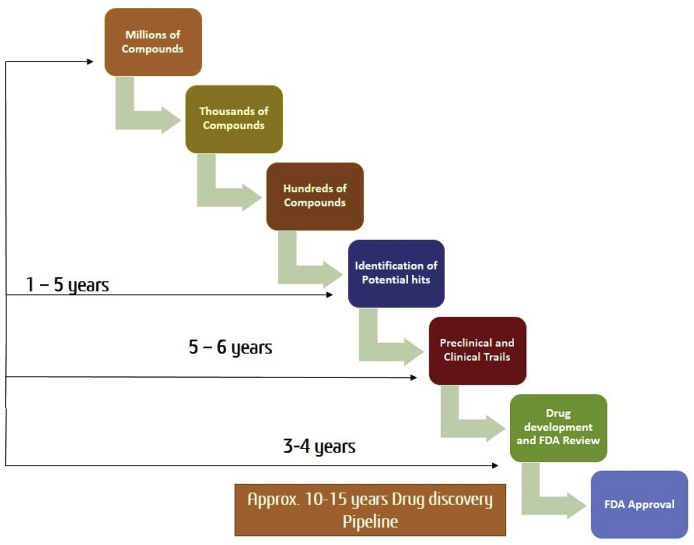
Expected drug discovery and development (DDD) timeline for COVID-19 infections.

Drug design and discovery (DDD) is a very complicated method that takes around 10–15 years for a drug to take in market for public use [[29], [30], [31]] (Fig. 4). Traditional DDD methods are not sufficient so that modern DDD methods were introduced to reduce the time and cost of novel DDD pipelines [32,33].

**Fig. 5:**
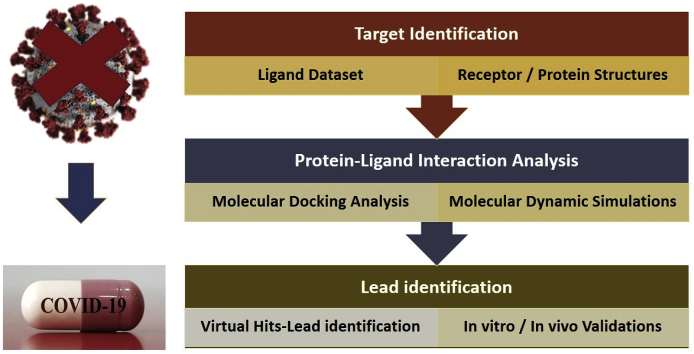
Frequently used computer-aided drug design (CADD) pipeline to identify or repurpose antiviral drugs against coronavirus disease (COVID-19).

Computer-aided drug design (CADD) is a popular method that signify the data from database and use softwares for predicting molecular structural properties of ligand and protein, predicting the activity/mechanism of unknown compounds on the basis of the known dataset with the help of applied mathematics and computational methods, and make the raw information as a significant outcome for the development of medicinal industry [32,34,35]. Advance computers are highly significant for the management of large datasets in modern DDD and open new ways for the development of novel therapeutics [32]. Computer-based/virtual screening (VS) technique is highly useful for screening of potential hits and then hit-lead compounds, based on the available data and advanced DDD methods, public repository of chemical compounds and protein targets have stuffiest information to lead a CADD project, these databases includes, PubChem database [36], CHEMBL database [37], ChemSpider database [38] National cancer institute open database compound [39], ZINC database [40], Protein databank (PDB) [41], PROXiMATE database [42], MIPS database [43] and many more [29,32]. Molecular docking (protein-ligand interaction analysis) and further atomic level analysis by molecular dynamic simulation are the frequently applicable methods of CADD to repurpose the novel and highly putative drugs against multiple life-threating diseases [44,45]. It explains the behavious of small chemical entities in the active sites of selected protein/receptor, and helpful in determining the activity of selected checmials [46,47]. Many advance tools has been discovered for easy docking proceudres such as Autodock Vina [44], molecular operating environement (MOE) [48], Gold [49], Glide [50], LigandFit [51], and FlexX [52] and for molecular dynamic simulation, most important tools are Amber [53], Gromacs [54], and Desmond [55]. There are many studied supported with molecular docking and molecular dynamic simulation studies for COVID-19 disease and reproposed new drugs based on the available antiviral drugs dataset [56,57,58]. Currently frequently acceptable CADD pipeline to identify or repurpose antiviral drugs against coronavirus disease (COVID-19) is shown in Fig. 5. Detail CADD-based studies for natural drug discovery for COVID-19 has been discussed in this review.

## 4 Phytomedicine identified by computer-aided drug design (CADD) techniques for COVID-19 disease

Several attempts have been made to investigate the repurposing of current antiviral treatments and to test traditional herbal medicines renowned for their health-promoting and immune-boosting properties against SARS-CoV-2. The phytochemical Cyanin **(1)** isolated from Zingiber officinale displayed broad-spectrum inhibitory efficacy against the major proteases of SARS-CoV-2, SARS-CoV, and MERS-CoV, with binding energies of −8.3 kcal/mol, −8.2 kcal/mol, and −7.7 kcal/mol, respectively [59].

The phytochemical dataset is constructed using a molecular docking method based on probability that distinct phytochemicals interact with the primary protease. In the docking analysis, the best performers, Medicagol **(2)**, Faradiol **(3)**, and Flavanthrin **(4)**, exhibited binding scores of −8.3, −8.6, and −8.8 kcal/mol, respectively. In addition, numerous characteristics derived from the molecular dynamics simulation validated the stability of the docked complexes. In addition, absorption, distribution, metabolism, excretion, and toxicity (ADMET) research indicated that none of the chemicals examined were hazardous or carcinogenic [60].

Using molecular docking and molecular dynamics (MD) simulations, an attempt was made to identify natural phytochemicals from therapeutic plants so that they may be repurposed against COVID-19. Molecular docking analysis revealed a number of potential inhibitors of SARS-CoV-2 Mpro (Main protease), including Withanoside V **(5)** and Somniferine **(6)** from *Withania somnifera* (Ashwagandha), Tinocordiside **(7)** from *Tinospora cordifolia* (Giloy), and Vicenin **(8)**, Isorientin **(9)**, and Ursolic acid **(10)** from *Ocimum sanctum* (Tulsi). Predictions from ADMET studies indicated that the best-docked phytochemicals were safe and possessed drug-like effects [61].

Based on the affinity of molecular docking, four natural metabolites, namely Sesamin **(11)**, Sesaminol **(12)**, Sesamolin **(13)**, and Sesamolinol **(14)**, have been classified as the most strongly interacting molecules with M^pro^. In addition, the stability of all these sesame-specific natural compounds was assessed using 200 ns of molecular dynamics (MD) simulations. Simulations of molecular dynamics and estimations of free energy indicated that these compounds have stability and favourable energies, resulting in strong interaction with M^pro^ [62].

Using molecular docking, six naturally occurring compounds with anticancer characteristics were screened (Ellipticine **(15)**, Ecteinascidin **(16)**, Homoharringtonine **(17)**, Dolastatin **(18)**, Halichondrin **(19)**, and Plicamycin **(20)**. Docking findings have conclusively demonstrated ligand binding to the SARS-CoV-2 RdRp protein. These findings give a rationale for repurposing and employing compounds derived from plants and animals as prospective treatments for the coronavirus disease 2019 (COVID-19) infection, since they may be effective therapies for the same [63].

As part of a technique based on molecular simulation, the inhibitory action of thirty chemicals extracted from the plant *Aerva lanata* was described. On the principal protease (PDB:6Y7) of SARS-CoV-2, the expected activity of the tested ligands was comparable to that of the current antiviral agent, hydroxy chloroquine (HCQ) **(75).** Some phytochemicals, such as Arvoside **(21),** which is unique to *Aerva lanata*, are predicted to have very significant *anti*-coronavirus activity due to their potency [64].

### 4.1 Glycosides

Yang et al. examined the chemical components of QFPD (QFPD is an off-the-shelf product used to treat COVID-19) in the treatment of COVID-19 [65]. Toll-like signaling pathway inhibition was responsible for the sickness inhibitory effect of the decoction on the COVID-19 illness network. Glycyrrhizic acid **(22)** and Ephedrine **(23)** were found in the decoction in various amounts. While investigating the efficacy of a rat model of pneumonia, the transcriptomic analysis showed that the decoction regulated the coagulation system during the inflammatory state, which helped to enhance the QFPD's capacity to intervene in the inflammatory storm generated by COVID-19.

Rutin (24) and Nicotiflorin (25) are glycoside flavonoids found in various medicinal plants and ayurvedic tea [63]. Rutin Hydrolysis Quercetin and other human metabolites have antioxidant, anti-inflammatory, and antiviral characteristics that contribute to a healthy diet. Rutin (24) and Nicotiflorin (25) are deglycosylated for the investigation. These compounds take on plasma's glucuronate and sulphate. Quercetin (26) and Kaempferol sulphates (27) may be SARS-CoV-2 3CLpro and RdRp inhibitors. Quercetin-3-O-glucuronide (28), a fluorinated flavonoid glucuronide, is a 3CL pro inhibitor.

Flavonoid sulphates can fight HIV and herpes simplex, according to studies. Using the latest postmortem study, COVID-19 is now accessible to treat Brazilians with coagulum intravascular dissemination (CID). It contains anticoagulant, anti-inflammatory, and possible protective properties against acute lung damage. Rutin (24) has activity in the therapy of 3CL pro and RDRP-SRS-CoV-2 (ALI). Rutin (24) injected intravenously or intranasally enters the bloodstream quickly with minimal digestion.

Some natural items that contain Rutin **(24)** include oranges, asparagus honeycomb onions, green tea figs, and most citrus fruits. Rutin **(24)** showed the highest binding affinity to the receptor with 8.8 kcal/mol and 0.336 M [66].

Calendula officinalis has the highest phytochemical binding energy score of 0.398 μM (Kcal/mol and Kd) [65]. Isorhamnetin-3-o-b-D interacts with amino acid residues except N3, which has no analogue. We can expect Isorhamnetin-3-O-glucoside **(29)** to interact with all essential amino acid residues that inhibit the receptor protein from recognizing the molecule. 11 of Narcissin's 13 interactions with amino acids were notable. Narcissin **(30)** may be an inhibitor that interacts with primary amino acid residues. Calendula glycoside B **(31)** binds to the receptor with an affinity of 8.2 kcal/mol and a dissociation constant of 0.928 μM 14 amino acid residues bind similarly to the natural ligand.

In silico, researchers predicted the toxicity of the Lauruside **(32)** candidates in the current study by using the programme ProTox (tox.charite.de) and found that all of them are assigned to toxicity class IV, which means they are predicted to have an oral lethal dose (LD50) in rodents of 2500 mg/kg[67]. On the other hand, Kaempferol **(33)**, a tetrahydroxyflavone with an affinity for Mpro that is anticipated to have class V toxicity, is thought to have a more significant biosafety potential even though its affinity for Mpro is lower than that of Lauruside **(32)**. This may result in lowering the binding energy of our complexes, which will lead to a greater affinity.

Glycoproteins like M^pro^ and Spike RBD related to ACE2 inhibition are tested for various compounds, including “coumarins, terpenoids, flavonoids, phenols, glycosides, and polyphenols, catechins, etc” [68]. Many flavonoid categories were studied for flavonol glycosides, such as Rutin **(24),** Astragalin **(34),** Quercetin **(26),** Baicalin **(35)**, Miricetine-3-glucoside **(36)** amentoflavone, etc. Binding affinity to flavones (Apigenin **(37),** Chrysin **(38),** and Luteolin **(39)** and soybean isoflavones were shown to be moderate (Daidzein **(40)** and Genistein **(41)**). Additionally, high binding of certain flavan-3-ols (catechins/procyanidins) and multifaceted oligomeric procyanidins like A3, A4, A1, and B3 had a wide range of binding values, Cordifoliside D **(42)**, a furanoid diterpene glycoside from *Tinospora cordifolia*, showed a strong *anti*-Mpro value. Include Anthraquinones **(43)**, Hypericin **(44)**, and Emodine 8-glucosides **(45)** as possibilities for M^pro^. One of the biggest substances concerning connecting with M^pro^ and RBD spike is a steroid glycoside known as Solanine **(46)** and other compounds like Rutin **(24)** and Acetoside **(47)**. Curcumin **(48)** and Acetoside **(47)** in Curcumin **(48)**, and Acetoside **(47)** also block M^pro^ covalently. The properties of lipophilic Curcumin **(48)** are superior to lipophilic lysine as well as those of lipophilic streptozotocin, According to Lipinski's 5th criteria. Moreover, ligand-receptor complexes “Curcumin **(48)** with Mpro, Solanine **(46)** with Mpro, and spike RBD” formed time-dependent complexes throughout 10 ns in the MD simulation.

Except for flavonol G9, practically all discovered flavonols (G1–G11) showed excellent binding stability in the region of N3 binding compared to covid-19 main protease inhibitor “Darunavir (49)" 68. Action requires rutinose (3-l-rhamnopyranosyl-(16)-d-glucopyranose). Rutinosides G5, G7 demonstrated better receptor interaction than monoglucosides G9, G11. Less active than robinbiosides G6, G8 were rutinosides G5, G7 (galactose replaces the glucose component of rutinose). Rutinosides G5, G7 were more active than G3, G1. A flavonol's ring B ortho methyl group reduces aglycone binding stability. Isorhamnetin monoglucoside G9 has a comparable glycone but a distinct non-sugar moiety.

### 4.2 Alkaloids

The spikes of SARS-CoV-2 were displayed to bind with ACE2 in glycoprotein's receptor-binding domain. Alkaloids interacted with the receptor-binding domain of SARS-CoV-2 spike glycoprotein, inhibiting receptor attachment [69]. SARS-greater CoV-2 binds to human ACE2 through amino acid (Gln 493 and Glu 484) mutagenesis. They should also get combined to ACE2's active site present, but not the viral spike glycoprotein binding site. However, the protein's three-dimensional shape has changed, altering regions required for spike protein binding. The ACE2 inhibitor inhibits SARS-CoV spike protein-mediated cell fusion. SARS-CoV-2 has a host receptor, thus this may happen (ACE2). 18 alkaloids showed higher binding energies than Lisinopril **(50)**, a common ACE2 inhibitor. Secondary metabolites of *Cladosporium* sp. PJX-41, an *anti*-H1N1 fungus from mangroves both Norquinadoline A **(51)** and Deoxynortryptoquivaline **(52)** are possible SARS-CoV-2 major protease inhibitants.

Garg et al. found that Thalimonine **(53)**-Mpro had the highest binding energy (−8.19) [70]. CYS145 generated 2-Hydrogen bonds with SER144 through Pi-donor and Alkyl interactions. HIS41 has two electrostatic benzene rings. HIS41 also has Pi-alkyl interactions. Both sulfur-containing residues, MET165 and MET49, established sulfur bonds. “HIS164 and ASP187” establish a π-Hydrogen-bond with ligand. Sophaline D **(54)**-Mpro docked complex has −8.8 kcal/mol of binding energy. Well-known interactions include H-bonds, Alkyl, Alkyl-π-Alkyl, C–H bonds, and Vander Waal attractions. HIS163 bonds with other residues. HIS41, CYS145, MET165 form pi-alkyl linkages. C–H 12 van der Waals residues stabilize HIS164. Tomatidine (55)-Mpro binding energy is −9.58 kcal/mol. HIS164 has one hydrogen bond. GLN189 has 1 C–H bond. Ring-structured “HIS41, HIS163, and HIS172” formed π-alkyl bonds with Tomatidine (55). There were 8 van der Waals interactions in the protein-ligand complex. Both domains contribute one residue to substrate binding. SARS-CoVs share His41 and Cys145. His41 is the base and Cys145's –SH group is the electrostatic trigger in the Cys-His dyad. Mpro needs a catalytic dyad to transform substrates. Minor residue alterations inactivate enzymes.

The alkaloids' *anti*-coronavirus activity is related to 3CL^pro^ suppression [70]. SARS-CoV-2 3CL^pro^ inhibitory potential of various alkaloids like “Cryptospirolepine **(56), 10-**hydroxyusambarensin**e (57)** and Cryptoquindoline **(58)**,” these all are the most docked alkaloids. The strongest interactions were with SARS-CoV and MERS-CoV 3CL^pro^, whereas 10-hydroxyusambarensine **(57)** had the least. SARS-CoV-2 and SARS-CoV shared top docked terpenoids binding. Its ability to get attached to the 3CLpro moiety of SARS-CoV-2 and SARS-CoV crafts it an effective antiviral. Inhibitors of MERS-CoV 3CL^pro^ that bind to an amino acid residue in domain III include iso guesterin and 20-epibryonolic acid. Some inhibitors might also target 3CL^pro^ domain III. The terpenoids' greater interactions with 3CL^pro^ than reference chemicals suggest they alter viral protease activity. They were utilised as receptor-binding residues together with Gln189 and His 164. The CysHis catalytic dyad (Cys145 and His41) and “HIS163/HIS172/GLU166” exhibited similar ligand-binding patterns to ritonavir. It also demonstrates that SARS-Cov-2 3CL^pro^ inhibitors may impede SARS-Cov-3CL^pro^ activity. “3CLpro of MERS-CoV” binds to its highest, most docked ligands uniquely [70].

Ghosh et al. used the CoV-2 M^pro^ variant to investigate the binding characteristics of two anti-viral medicines (“Lopinavir **(59)** and Darunavir **(49)**; binding affinity −7.3 to −7.4 kcal/mol”) and alkaloids anisotine derived from *Justicia adhatoda* [71]. Alkaloid (anisotine) showed a high affinity with energy value of −8.0 kcal/mol for both Mpro catalytic residues (His41 and Cys145).

The study used molecular docking to search for direct exchanges between “SARS-CoV-2 RNA-dependent RNA polymerase” (RdRp) and a large number of bioactive chemicals obtained from African medicinal plants [72]. They were docked against the active sites of different variants of CoV-2. Docking studies of SARS-CoV-2 RdRp were accomplished with the active site of eight different conformations derived through MDS system equilibration using the hit list of seven chemicals. Stychnopentamine **(60)**, Cryptospirolepine **(56),** and Usambarensine **(61)** were used as positive controls (12-epi-millettosin). It is possible to construct an *anti*-coronavirus cocktail using phytochemicals, Remdesivir **(62)**, and Sofosbuvir **(63).**

Docking phosphorylated rdRp and rdRpol was accomplished with RdRp, M^pro^, and RdRpol to find probable protein-alkaliod relative interactions. The binding energies of thirteen alkaloids were obtained in the values between −6.9 and −10.6 kcal/mol. The best binding affinities were observed in Cryptomisrine **(64)** and Biscryptolepine **(65).** Cryptomisrine **(64)** and Cryptospirolepine **(56)** intermingled to Mpro ligands like His41 and Cys145. The groups like π-alkyl, π-π T-shaped sulfur, and π-π stacked hydrophobic bonds were involved. Cryptomisrine **(64)** quinoline nitrogen and Mpro's Met165 formed hydrogen bonds at 2.88 at bond angles of 116.3° and 103°. (DHA and HAY, respectively). In the mid of the carbonyl oxygen and 143 Gly (Fig. 2) at 2.41 and angles 142.895° (DHA and HAY), but between Asn 142 and the carbonyl oxygen at 2.17 and angles 131.604° and 138.885° (DHA and HAY). Cryptoquinoline and Biscryptolepine **(65)** were pi-alkyl hydrophobic with Met165 and Met49, but pi-sulfur and anion with Glu166 [73].

For the guanidine alkaloids that include two main glycoproteins named spike (PDB ID: 6VYB), and membrane glycoproteins (PDB ID: 6M17) a phosphoprotein nucleocapsid (Protein Database ID: 6VYO), M^pro^ (6lu7) and a non-conformational protein (nsp10), the study done by Demerdash et al. was conducted (PDB ID: 6W4H) [74]. Crambescidin 786 **(67)** and Crambescidin 826 **(68)** stand out because of their pentacyclic alkaloids. Compound 5 has a strong affinity for Mpro with a G value of 8.05 kcal/mol, nsp10 with a G value of around 9.06 kcal/mol, and a phosphoprotein called nucleocapsid (G = 6.39 kcal/mol). Compound 13's affinity for the following chemical affinity groups was found as 8.0 kcal/mol (M^pro^), 7.0 kcal/mol (spike glycoproteins), and 8.01 kcal/mol (nucleocapsid phosphoprotein). Most of the compounds also behaved as agonists of CYP2D6, non-toxic to liver, and bind to the plasma proteins with a proportion that is not as much of 90%.

In silico investigations were performed on 97 antiviral alkaloids from African medicinal plants [75]. Docking for alkaloids was performed against any spikes glycoprotein or ACE2, receptor, or TMPRSS2. The topmost of all twenty alkaloids were tested for interactions with CoV-SARS and CoV-MERS spike glycoproteins and the ACE2-CoV-2-SARS. Due to their elevated binding affinity, firmness of alkaloidal protein complexes, and amino acid bonding with key protein binding hotspots, Cryptospirolepine **(56)**, 10-hydroxyusambarensine **(57)**, and Cryptoquindoline **(58)** have the potential to modify SARS-CoV-2 membrane facilitated host cell entry.

**Fig. 6:**
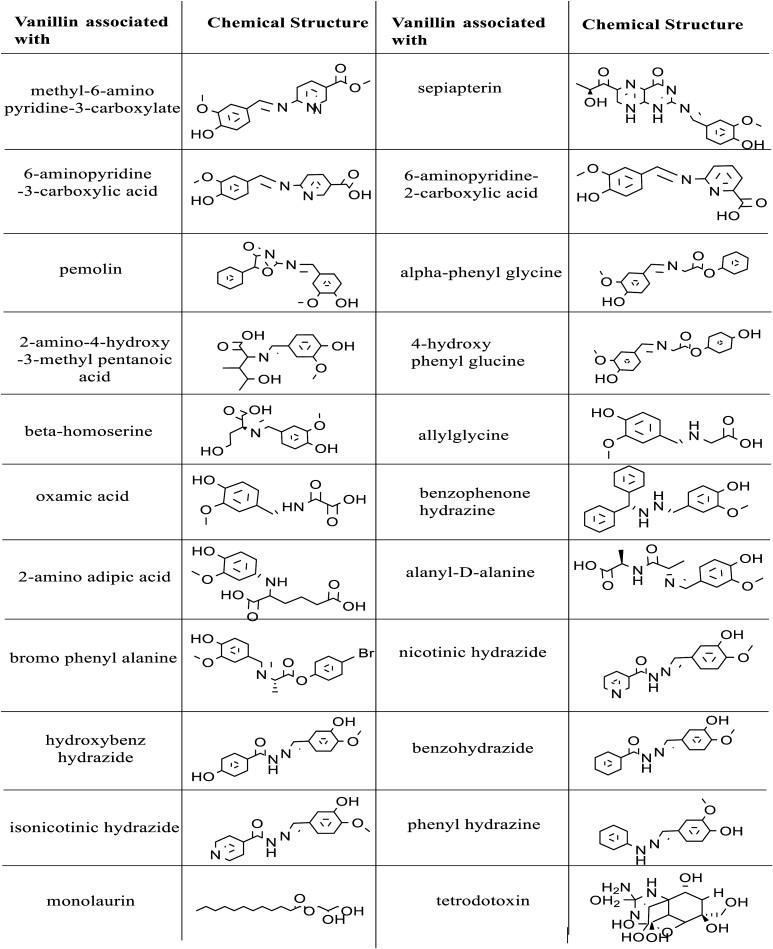
Structural representation of various vanillin derivatives studied by Law et al. Ref [34].

The training set pharmacophore model has four common features: two Hydrogen Bond Acceptors (HBA) and two Hydrogen Bond Donors (HBD) [34]. Every single derivative of Vanillin **(66)** and Monolaurin **(69)** shared collectively 3, 4 structural characteristics, suggesting they may be SARS-CoV-2 inhibitors. Sepiapterin **(70)** had the most significant alignment score (47.04), followed by β-homoserine and allylglycine (45.39). Both Monolaurin **(69)** and Tetrodotoxin **(71)** had alignment scores of 43.39, with four comparable pharmacophore characteristics aligned with hydroxyl functionality, indicating that both can act as prospective inhibitors of MPro. Utmost twelve Vanillin **(66)** derivatives have three characteristics. They range between the values 36.41 and 38.33, in which Vanillin **(66)** is linked to pemoline, securing a maximum alignment score of 38.33. These derivatives employing oxazole and aminopyridines moieties united finely with the heterocyclic structure, pyrimidine ring present there and showed the HBA characters with active sites. “HOH671A, HOH741A”, while the aromatic ring displayed a different feature, i.e., HY with active site (LEU58A). Because the studied **Vanillin (66)** compound fit well with the ligand and have similar pharmacophore characteristics, they are shown to be promising M^pro^ inhibitors of CoV-2 virus *in silico,* as shown in Fig. 6.

A negative value for the global energy of interacting molecules suggests a greater binding probability [76]. According to molecular docking research, Caulerpin **(72)** has the most antiviral efficacy against CoV-2 protease, spike proteins of SARS-CoV-2, and ACE2. Researchers investigated the use of the highest binding energy antiviral medicines like Lopinavir **(59)** and Simeprevir **(73)** in conjunction with Caulerpin **(72),** and it was shown to spoil the stability of spike glycoproteins, protease of CoV-2 and also host ACE2 receptors. It is proposed to use it in conjunction with medicines like Lopinavir **(59)** and Simeprevir **(73)**, which treat COVID-19 patients. Caulerpin **(72)** had a greater binding energy to all investigated receptors than known drugs like Simeprevir **(73)**, Hydroxychloroquine **(74),** Lopinavir **(59)**, Chloroquine **(75),** and Amprenavir **(74)**. Complementing to the conventional antiviral drugs, Caulerpin **(72)** may be a potent antiviral molecule for disrupting the integrity of SARS-CoV-2 protein receptors. Compared with other medications, Simeprevir **(73)** and Lopinavir **(59)** displayed the greatest bond energies for 6LU7, 6VYB, and 1R42. It seems that “Simeprevir **(73)**, Lopinavir **(59)**, and Caulerpin **(72)**” connected distinct amino acid molecules in combination and treatment. As a result, Caulerpin **(72)** combined with Simeprevir **(73)** and Lopinavir **(59)** proved to be extremely effective against SARS-CoV-2, and these medications may be repurposed to inhibit COVID-19.

Borkotoky and his co-workers studied chemical components of Neem (Azardica Indica), molecular dynamics modeling, and virtual screening to find small compounds that bind to SARS-CoV-2 structural proteins M and E^77^. The M-protein part interacted with almost all key component proteins named N, S, and E proteins. The protein E's, an ion channel protein, played a role in contacting membrane, and winding is required for viral budding [78]. Since the E-ion-channel protein's activity is needed for viral replication. Thus antagonists aiming at the E-protein site may assist limit virus generation [13]. The anti-Influenza medication amantadine also binds to E-protein ion channels, but the impact is mild [79]. These chemicals interact with cross membrane area residues which are Asp 15 (N15) and Val 25 (V25), to affect the protein's biological function (V25). Previous research has demonstrated that these mutations (N15A & V25F) impair ion channel function and oligomerization of SARS CoV E protein [[78], [79], [80], [81]]. When Neem compounds are docked, displayed two potential sites of binding for M-protein. The C-terminal 20 residues of “Nimocin and Nimbolin A″ are connected to the cross membrane region. The MERS-CoV *M*-C-terminal protein's tail, comprising Lysine 199, Glycine 201, Tyrosine 203, and Arginine 204, is essential for TGN localization [82]. After a simulation study revealed that “Glycine 202 and Tyrosine 204” contribute significantly to the binding free energy of M-protein [77].

Using pharmacophore modeling, auto-QSAR prediction, and molecular investigations, researchers have compared nine flavonoids from three different therapeutic plants: *Tithonia diversifolia, Blighia sapida, and Irvingia gabonensis,* with commonly used medicines [83]. Notably, the Auto QSAR method predicted the compounds' pIC 50 values using machine learning. Lutein **(75)** scored highest in affinity with the energy of −9.60 kcal/mol, next accompanied by “Apigenin **(37)** and Ellagic acid **(76)**” with the values of −9.6 and −9.5 kcal/mol, of each one. Gallic acid has the minimum binding energy (−6.30 kcal/mol). Docking scores for Donepezil **(77)** and Galanthamine **(78)** were −10.40 and −7.93 kcal/mol, respectively. It was determined that no flavonoids were poisonous and had acceptable assimilation characteristics for the objectives studied.

### 4.3 Terpenoids

Giofre et al. tested fourteen different limonoid and terpenoid compounds for their potential to hinder SARS-CoV-2 remedial target proteins [47]. In molecular dynamics simulations, Nomilin **(79),** Deacetylnomilin **(80)** and Ichangin **(81)**, and Amyrin **(82),** a terpenoid, had excellent binding energies with the SARS-CoV-2 virus. Deacetylnomilin **(80)** and Ichangin**(81)** interacted directly with the enzyme's catalytic dyad, indicating a function in inhibiting SARS-CoV-2 replication and expansion. The anticipated pharmacokinetic profiles and docking score suggested that these triterpenoids may also prove beneficial in antagonizing the effects of CoV-2 M^pro^ urging additional for *in vitro* and *in vivo* studies to fully understand and validate their inhibitory potential. For example, Deacetylnomilin **(80)** had an average RMSD value of 0.74, having an average binding energy of 66.581 kcal/mol with Mpro, whereas, Ichangin **(81)** had a high RMSD value of 1.56 and average interaction energy of 51.045 kcal/mol with Mpro, respectively. The interaction energy of Nomilin **(79),** on the other hand, was 54 kcal/mol, which indicated significant contact with M^pro^ cavity despite its 3.75 RMSD average. Amyrin **(82)** had the best results among the terpenoids, holding a mean value of RMSD as 1.34, also binding energy of 45.2 kcal/mol with M^pro^. The high RMSD values of oleanolic aldehyde and ichangensin and other terpenoids like limonin, obacunone, and tirucallol make them poor inhibitor candidates.

24-dimethylene cycloartenol **(83)** and Isoiguesterin **(84)** have been shown to aim at ACE2 receptor site and the virus interaction with the host. These molecules showed its capacity to get bound and inhibit communications of hotspot 31 residual groups [84]. The human ACE2 residues at different amino acid sites of lysine and tyrosine are critical for coronavirus S-protein binding. Because hotspots 31 and 353 are masked in a hydrophobic environment, interactions inside this area may influence substrate binding. In comparative research, five phytochemicals from Chinese and Indian herbs behaved differentially on the active site of ACE2, but all tended to disrupt the conformation required for its attachment with viral S protein. They bind to ACE2's Site-2 binding site similarly to drugs like delapril, Lisinopril, and perindopril.

The M^pro^ protein of CoV-2 was utilised for the discovery of covid inhibitor [85]. Any compound having 15% of the value of Mpro is likely to be observed as a good inhibitor of the molecule. Only A7 and some terpenoids (T1,2,3,4,10,14,16) have met the criteria [86]. T14 has a comparable binding ability to T3. Polyphenols and peptides get poor scores. The decarine plant Zanthoxylum ailanthoides root bark alkaloid A7 has strong HIV activity (EC50 0.1 mg/mL) [48.] The terpenoids known by the name T1 and T4 are derived from the aquatic source *Cacospongia mycofijiensis* [87]. Also, an anti-cancer representative with EC50 value of 17.9–20.6 M was discovered in apple peel [88]. The triterpene obtained from Ganoderma colossum inhibited HIV-1 protease with an EC50 value of more than 15.3 M [88]. Micafungin **(85),** a diterpene found in the mycolic cell wall, is accessible as an injectable antifungal agent [89]. They have different carbonyl functionalities like ketones, esters, and amides with *cis*-trans geometry. The unsaturated group was bonded covalently to the Cystine residue inside the enzymatic active site. The cis isomer of conjugated carbonyl (T2) destabilises the molecular arrangement (G0 value is 152 kJ/mol).

In contrast, the Z shape of the amide stabilises the arrangement (G0 value 24 kJ/mol) and destabilises the molecule on the whole. Like T3, the stiff E, Z double bonds in T14 favour their binding. Three-domain protease enzyme CoV M^pro^ cleaves polyproteins to generate mature proteins. Notably, the active site is located in a groove connecting domain I and II, which is structurally most preserved amongst CoV M^pro^ [90]. This site may covalently bind to inhibitor natural products including aldehyde groups, active ketone groups, and unsaturated carbonyl groups. The CoV M^pro^ is physically and functionally distinct from the human protease enzyme, making it an excellent antiviral target [91]. Potential drug candidates exhibited significant activity against COVID-19 disease identified by CADD are highlighted in Table 1.

**Table 1:** Potential drug candidates exhibited significant activity against COVID-19 disease identified by CADD applications.

Chemical name	Chemical Structure	Chemical name	Chemical Structure
Cyanin (1)		Medicagol (2)	
Faradiol (3)		Flavanthrin (4)	
Withanoside V (5)		Somniferine (6)	
Tinocordiside (7)		Vicenin (8)	
Isorientin (9)		Ursolic acid (10)	
Sesamin (11)		Sesaminol (12)	
Sesamolin (13)		Sesamolinol (14)	
Ellipticine (15)		Ecteinascidin (16)	
Homoharringtonine (17)		Dolastatin (18)	
Halichondrin (19)		Plicamycin (20)	
Arvoside (21)		Glycyrrhizic acid (22)	
Ephedrine (21)		Rutin (24)	
Nicotiflorin (25)		Quercetin (26)	
Kaempferol sulphates (27)		Quercetin-3-O-glucuronide (28)	
Isorhamnetin-3-O-glucoside (29)		Narcissin (30)	
Calendula glycoside B (31)		Lauruside (32)	
Kaempferol (33)		Astragalin (34)	
Baicalin (35)		Miricetine-3-glucoside (36)	
Apigenin (37)		Chrysin (38)	
Luteolin (39)		Daidzein (40)	
Genistein (41)		Cordifoliside D (42)	
Anthraquinones (43)		Hypericin (44)	
emodine 8-glucosides (45)		Solanine (46)	
Acetoside (47)		Curcumin (48)	
Darunavir (49)		Lisinopril (50)	
Norquinadoline A (51)		Deoxynortryptoquivaline (52)	
Thalimonine (53)		Sophaline (54)	
Tomatidine (55)		Cryptospirolepine (56)	
10-hydroxyusambarensine (57)		Cryptoquindoline (58)	
Lopinavir (59)		Stychnopentamine (60)	
Usambarensine (61)		Remdesivir (62)	
Sofosbuvir (63)		Cryptomisrine (64)	
Biscryptolepine (65)		Vanillin (66)	
Crambescidin 786 (67)			
Crambescidin 826 (68)			
Monolaurin (69)		Sepiapterin (70)	
Tetrodotoxin (71)		Caulerpin (72)	
Simeprevir (73)		Hydroxychloroquine (74)	
Chloroquine (75)		Amprenavir (74)	
Lutein (75)		Ellagic acid (76)	
Donepezil (77)		Galanthamine (78)	
Nomilin (79)		Deacetylnomilin (80)	
Ichangin (81)		Amyrin (82)	
24-dimethylene cycloartenol (83)		Isoiguesterin (84)	
Micafungin (85)			

## 5 Conclusion and future directions

It can be stated that SARS-CoV-2's transmission and occurrence generally depend upon the interaction between the human host's immunity and the genomic characteristics of the virus-containing ribonucleic acid. Also, other factors like genetics, gender, age, nutritional and physical characteristics play a significant role in determining the severity of the infection [92,93].

As the virus is spreading exponentially, it has become an alarming situation. The best possible strategy to cope with such an infection is to avoid direct contact, social distancing, early detection, and symptomatic treatment. Also, the use of computer-aided drug designing tools and various other protease repurposing strategies has been done to determine potential treatment target molecules to discover new inhibitors [46,94]. The evolution of such new techniques and drug molecules is the need of the hour to benefit and treat all the patients battling COVID-19. However, the lack of belief in the power of scoring functions to provide precise binding energies is the approach's greatest limitation. This is because some intermolecular interaction parameters, such as solvation effect and entropy change, are difficult to forecast precisely. Another significant obstacle in the realm of docking is stiff receptors. The shape of a protein might vary based on the ligand to whom it binds. As a consequence, docking conducted using a rigid receptor will contribute significantly to a single receptor conformation, resulting in many false negatives where the ligand was later discovered to be active. Lastly, the spectrum of action towards off-target proteins is rarely observed, even using computer screens, and can only be determined through animal and human trials. The existing gaps in identifying plant-based treatments as structural protein inhibitors, such as spike, envelope, membrane, and nucleocapsid, will be addressed by further study in this regard. There might be huge benefits to this study in the fields of epidemiology and treatment and prevention. Investments in time and money for phytomolecular research could have a direct and long-term impact on the security of the health protection regime for humans and animals due to the growing interest of the public and governments around the world in improving the immunity of humans and animals to lethal viruses, such as SARS-CoV-2.

## Ethical approval

This article does not require any human/animal subjects to acquire such approval.

## FUNDING

No funding.

## Registration of research studies

Name of the registry: Not applicable.
Unique Identifying number or registration ID:
Hyperlink to your specific registration (must be publicly accessible and will be checked): Not applicable.

## Consent

Not applicable.

## Author contribution

Isha Rani and Avjit Kalsi: Conceptualization, Data curation, Writing-Original draft preparation, Writing- Reviewing and Editing. Rupesh K. Gautam: Conceptualization, Writing-Reviewing and Editing, Visualization. Gagandeep Kaur, Pankaj Sharma, Sumeet Gupta: Data curation, Writing-Original draft preparation, Writing- Reviewing and Editing. Hitesh Chopra, Shabana Bibi, Syed Umair Ahmad, Inderbir Singh: Data curation, Writing-Original draft preparation, Writing- Reviewing and Editing. Manish Dhawan: Writing-Reviewing and Editing, Visualization. Talha Bin Emran: Conceptualization, Writing-Reviewing and Editing, Visualization.

## Guarantor

Talha Bin Emran, Ph.D., Associate Professor, Department of Pharmacy, BGC Trust University Bangladesh, Chittagong 4381, Bangladesh. T: +88-030-3356193, Fax: +88-031-2550224, Cell: +88–01819942214. https://orcid.org/0000-0003-3188-2272. E-mail: talhabmb@bgctub.ac.bd.

## Data availability statement

The data that support the findings of this study are available from the corresponding author upon reasonable request.

## Declaration of competing interest

The authors declare that they have no conflicts of interest.
